# Size-dependent characteristics of electrostatically actuated fluid-conveying carbon nanotubes based on modified couple stress theory

**DOI:** 10.3762/bjnano.4.88

**Published:** 2013-11-20

**Authors:** Mir Masoud Seyyed Fakhrabadi, Abbas Rastgoo, Mohammad Taghi Ahmadian

**Affiliations:** 1 Karaj Branch, Islamic Azad University, Karaj, Iran; 2 School of Mechanical Engineering, College of Engineering, University of Tehran, Tehran, Iran; 3 Department of Mechanical Engineering, Sharif University of Technology, Tehran, Iran

**Keywords:** carbon nanotubes (CNT), electrostatic actuation, fluid flow, modified couple stress theory

## Abstract

The paper presents the effects of fluid flow on the static and dynamic properties of carbon nanotubes that convey a viscous fluid. The mathematical model is based on the modified couple stress theory. The effects of various fluid parameters and boundary conditions on the pull-in voltages are investigated in detail. The applicability of the proposed system as nanovalves or nanosensors in nanoscale fluidic systems is elaborated. The results confirm that the nanoscale system studied in this paper can be properly applied for these purposes.

## Introduction

Nanotechnology can be defined as the science of manipulating materials on an atomic or molecular scale [1]. Hence, it generally deals with investigating different aspects of the materials in atomic and molecular dimensions, which include chemical, physical, thermal, electrical and mechanical properties, in order to develop some novel systems and structures that can improve the quality of our daily lives. For example, many scientists all over the world are studying the medical applications of nanostructures in order to cure diseases such as cancer [2–4]. On the other hand, many researchers are making attempts to invent nanomaterials or nanomaterials-based micro/macro-materials, which can be advantageous in engineering applications [5–8]. It is worth noting that the research fields in nanotechnology are generally multidisciplinary.

One of the most important types of theses multidisciplinary fields are nano-electromechanical systems (NEMS). They include aspects of mechanical engineering, electrical engineering and some basic sciences. In general, NEMS are nanostructures such as beams, shells, plates, tubes or other similar structures, which are sensed or actuated by using electrical mechanisms. They can be applied as nanoswitches, nanocapacitors, nanotransistors and computer storage media. These systems have been studied by using both theoretical and experimental techniques. Of course, the former is more extensive, probably because of the higher costs of experimentation and less elaborated fabrication technologies for nanotechnology. Thus, numerical simulations that include discrete and continuum modeling techniques are conducted more often. In spite of the higher accuracy of discrete modeling approaches such as molecular dynamics, it is rarely applied in scrutinizing NEMS because of their limitation in analyzing the complicated behaviors of such systems [9–12]. Hence, continuum modeling is more frequent and is also applied in this paper. The elasticity equations, which govern the mechanical behaviors on the micro/nano-scale and the macro-scale, are very similar. However, one of the main differences is the appearance of some interatomic effects on the nanoscale such as van der Waals (vdW) interaction and/or the Casimir effect, a quantum effect that results from vacuum fluctuations.

**Figure 1:**
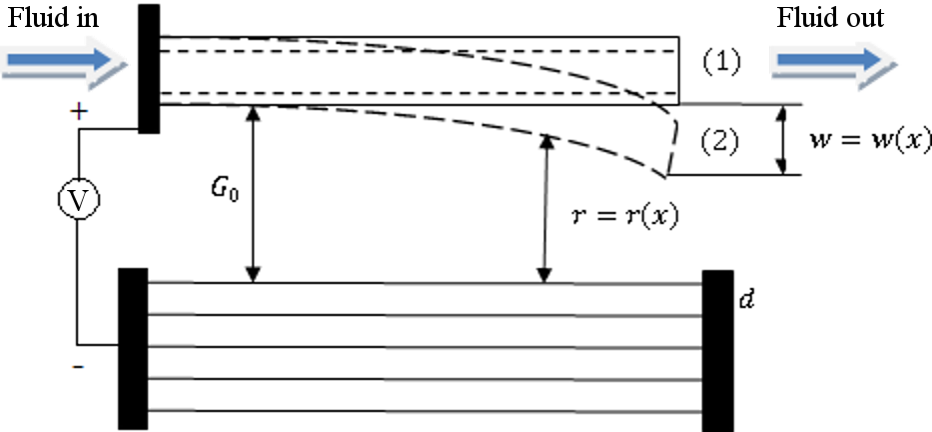
Application of an external force on a nanotube and its local buckling.

Among the various materials and structures made from different metallic and non-metallic materials, carbon nanomaterials play a special role. For instance, carbon nanotubes (CNTs) possess extraordinary chemical, physical, mechanical and electrical properties. Thus, since their discovery in 1991 by Iijima [13], they have attracted a lot of scientists and researchers all over the world to study their characteristics as well as their actual and potential applications. A mathematical formulation of the applicability of CNTs in NEMS was conducted by Dequesnes et al. [14]. They applied a model with one degree of freedom in order to study the manipulation of CNTs by using electrostatic actuation and vdW interactions. The results revealed that the vdW force played an important role in the deflection and pull-in behaviors of the CNTs. In electrostatic actuation, a voltage is applied to two electrodes with a gap in-between. In general, one of the electrodes is fixed and the other one is movable (Figure 1). The movable electrode is attracted towards the fixed one by the applied voltage. It is worth noting that the movable electrode cannot tolerate this attractive force up to any arbitrary value. The maximum threshold is known as the pull-in voltage and is to be discussed in detail in the following sections. In another paper, Dequesnes et al. [15] studied the static pull-in behaviors of the CNTs as well as their natural frequencies under electrostatic actuation, and the vdW interactions and compared some of the results with those obtained from molecular dynamics. The main difference between [14] and [15] is the more detailed formulation of vdW force between the CNT and graphene sheets. In the former, the researchers only studied the attractive term whereas in the latter they investigated the repulsive term as well.

Ke et al. further developed the studies of Dequence et al. by presenting two papers regarding CNT-based NEMS [16–17]. The first paper focused on the quality of the charge distribution and the second one was about the stretching effects on the doubly clamped CNTs. They also presented another paper regarding this field and validated the results obtained from modeling through experimental data [18]. In addition, Ouakad and Younis studied the dynamic behavior of the CNTs under electrostatic actuation and presented the frequency response of the systems as a function of different applied voltages [19]. Rasekh and Khadem added to the mathematical modeling of CNT-based NEMS more comprehensive results about the static and dynamic behavior of the CNTs under electrostatic and vdW forces [20].

Potential applications of CNTs range from nanoelectronic devices to nanofluidic systems [21–26]. Gases or liquids that flow through nanopipes or are confined to very tiny volumes are likely to find a wide variety of applications in nanotechnology, especially in nanomachines with moving parts. CNTs are excellent options for these purposes because of their extraordinary mechanical properties, their chemical and thermal stability, and their hollow geometries. For example, they can be used as hydraulic parts in support platforms or carry reactant molecules into reaction chambers [21]. Furthermore, CNTs have a potential usage as cancer therapy devices or nanovessels for conveying and storing fluids and for drug delivery [22].

Because of the potential application of CNTs to convey fluids, it is obvious that the effects of fluid flow on the mechanical behaviors of CNTs should be scrutinized by including them in the governing equations of fluid dynamics. The papers on NEMS mentioned above and also similar research only studied different aspects of the mechanical behavior of the nanostructures under electrostatic actuation but without considering fluids passing through them. Of course, the mechanical behavior of CNTs that convey fluids without electrostatic actuation was studied before. To our knowledge, Yoon et al. were the first to study the flutter instability that results from fluid flow in CNTs [23]. They presented the natural frequencies and the damping of the CNT for various flow velocities. Their work had some shortages, which were overcome by Lin and Qiao [24]. They applied the differential quadrature method and comprehensively investigated the mentioned phenomena. In another research, Wang and Ni studied the effects of the viscosity on the instability of the CNTs [25]. The authors of [23–25] applied the Euler–Bernoulli beam model in their researches. Chang and Lee used the Timoshenko beam model, which considers the rotational inertia and shear deformation, to investigate the effects of internal fluid flow on the transverse vibration [26]. They reported the natural frequencies of the system for different aspect ratios.

Despite some studies concerning the effects of fluid flow on the static and dynamic behavior of CNTs, the control of the flow was not investigated in detail. Only a limited number of papers reported rudimentary conceptual designs of CNTs as nanovalves [27–29]. It is clear that every flow system, even on the nanoscale, should have effective and applicable controlling devices such as valves, flow meters, pressure sensors, viscometers and densitometers. The development of such devices for the fluid flow through nanotubes is one of the main goals of this research.

Solares et al. designed a nanomechanical fluid control valve based on functionalized silicon cantilevers and modeled it by using molecular dynamics [27]. A settling of nanoparticles on the CNT resulted in its transverse deflection. Optimal design geometries and operational deflection ranges were estimated for a device that contained over 75000 atoms. It was shown that if the nanoparticles exceeded a maximum limit, bending and buckling would occur, and the fluid stream that passes through the nanotube would be blocked. In another research, Grujicic et al. applied this concept to boron nitride nanotubes as nanovalves [28]. Chen et al. designed a one-way nanovalve based on a CNT junction and a C_60_ molecule and simulated it by using molecular dynamics [29]. It was supposed that there is a flow through the CNT junction and a C_60_ molecule could block it inside the CNT. In spite of invaluable ideas behind the designs presented in [27–29], their extremely slow responses may impede a reliable performance in real-world systems, especially in those requiring fast actuation. This paper tries to develop a more applicable way to apply CNTs as devices to control fluids on the nanoscale. Details will be discussed in the following sections.

**Figure 2:**
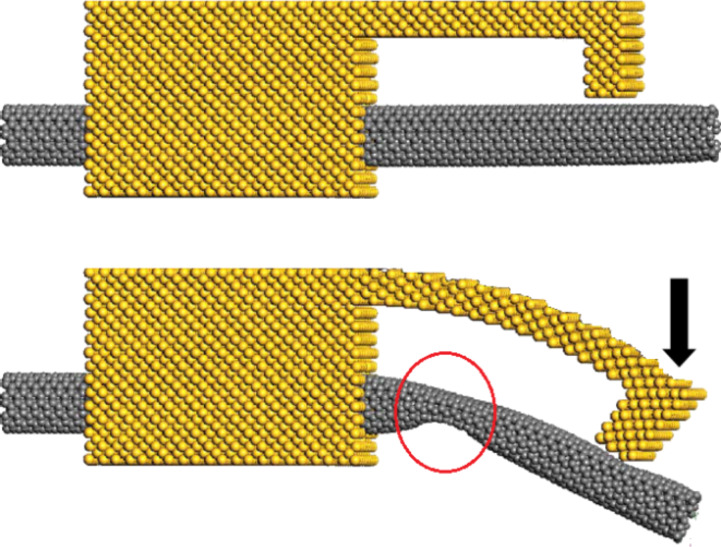
Electrostatic actuation of a CNT that conveys a fluid (top: “open”, bottom: “closed).

Electrostatically actuated CNTs have various potential applications. For example, in the static actuation, they could be used as nanovalves. It is shown in Figure 2, that if an external transverse force is apllied to a constrained CNT, a critical section deforms so that it confines the fluid [9,28]. The level of confinement is a function of the applied force and the resultant deformation.

In this paper, the effects of a fluid flow on the static and dynamic pull-in instabilities of the CNTs with cantilever and doubly clamped boundary conditions are investigated. The pull-in voltages of the CNTs for various fluid parameters including fluid viscosity, velocity, pressure and mass ratio (fluid mass/CNT mass) are scrutinized. A brief discussion on the buckling loads, vibrational behaviors and natural frequencies of the CNTs is presented.

## Description of the system

As shown in Figure 1, it is supposed that a CNT is suspended over graphene sheets with an initial gap *G*_0_ (state 1 in Figure 1). Continuum mechanics are applied in order to analyze the nanosystem. A voltage *V* is applied between the CNT as the positive electrode and the graphene sheets as the negative electrode (ground plate). This voltage and cooperated with the interatomic forces between the electrodes lead to deflection of the CNT towards the ground plate (state 2 in Figure 1). The deflection value, *w* = *w*(*x*), corresponds to the applied voltage up to the point at which the elasticity of the CNT can no longer compensate the forces that result from the applied voltage and the interatomic forces. Then, the CNT suddenly drops on the ground plate. This phenomenon is called pull-in instability and the corresponding voltage is the pull-in voltage. If the behavior is static, the pull-in will be static. In the case of dynamic motion, the pull-in will be a dynamic pull-in. Details are to be discussed in the following sections.

**Figure 3:**
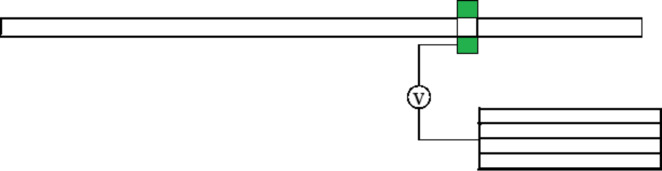
Connection between the nano sensor and larger tubing.

The most appropriate, and perhaps the only, method to effectively actuate CNTs at the nanoscale is the electrostatic actuation technique. The described external force in Figure 2 can be applied by the electrostatic actuation. Hence, the CNT can be utilized as a fast and more controllable nanovalve by using electrostatic actuation. On the other hand, the studied system can be applied as a sensor to sense and measure the fluid velocity, viscosity, pressure and density. Altering these fluid parameters results in changes in the static and dynamic pull-in voltages. Both the static and the dynamic pull-in values can be used to obtain certain values of the mentioned fluid properties. Some studies applied other criteria for this purpose such as a shift in the natural frequencies [30], but it is clear that the method presented in this paper is more applicable and measurable. At this stage, the concepts in this paper are merely theoretical. At a later point, a fabrication process may be considered. The nanosystem should be connected to a larger tubing. One of the applicable techniques is applying the system at the end of the larger tubing. This means that a part of the larger tube can be the movable part of the system as considered in this paper, and the other parts such as graphene sheets and actuation devices can be added to the considered section of the nanotube (Figure 3).

Another application of the system is its utilization for feedback control. A control strategy should have some control parameters that an algorithm adjusts in order to control the system. Passing viscous fluids through the CNT with different fluid characteristics can provide a platform for controlling the electromechanical behaviors of the CNT under electrostatic actuation by imposing changes in the stiffness and damping ratios, as will be studied in the paper.

## Mathematical formulae

All mathematical formulae and expressions that are used in this study can be found in Supporting Information File 1.

## Results and Discussion

**Figure 4:**
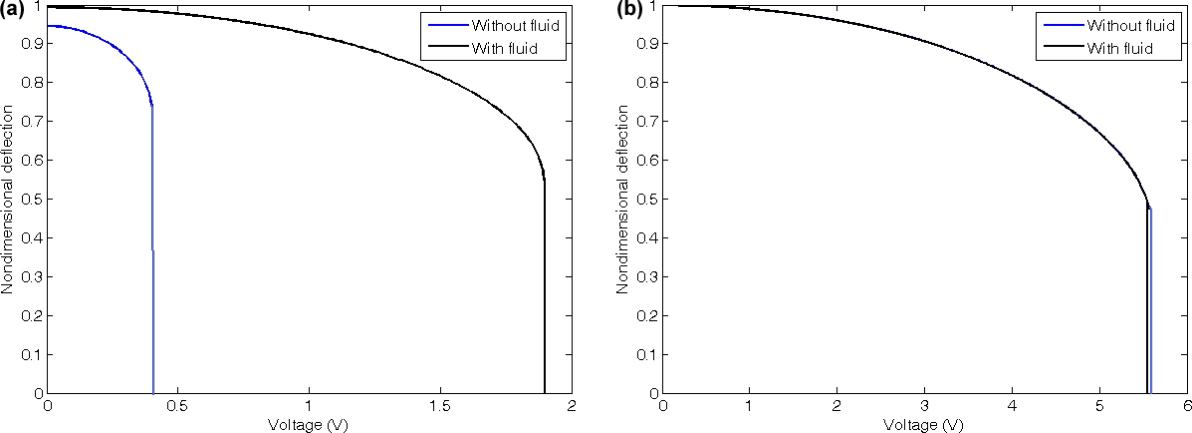
Effects of fluid flow on static pull-in of the (a) cantilever and (b) doubly clamped CNT under static DC voltage.

The length and chirality of the CNT considered in this paper are 50 nm and (10,10), respectively. Based on the chirality, the diameter of the CNT is considered to be about 1.4 nm. The effects of different dimensions on the pull-in behavior of CNTs were studied in our previous papers. In this research, we investigate the effects of fluid flow on the static and dynamic pull-in instabilities of the CNTs with cantilever and doubly clamped boundary conditions by using MCST. Figure 4 shows the static pull-in phenomena for both cantilever and doubly clamped CNTs. The figure reveals that the CNT deflects under the electrostatic actuation up to a maximum limit and suddenly drops on the ground plate after it. The reference fluid velocity equals v* = *L*/t* and the parameters of fluid flow of Figure 4 are given in Table 1. The presented properties remain constant unless the cases stated explicitly or the cases that their variations are investigated.

**Table 1:** Viscous fluid flow parameters of Figure 4.

flow parameter	numerical value

dynamic viscosity	μ = 1 Pa·s
pressure	*P* = 1 kPa
non-dimensional fluid velocity	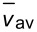 = *v*_av_/v* = 1
mass ratio (fluid mass/CNT mass)	*m*_r_ = *m*_f_/*m*_c_ = 0.1
non-dimensional length scale parameter	l* = 0.1

Figure 4 shows that the effects of fluid flow on the cantilevered CNTs are remarkably stronger than those on the doubly clamped CNTs. The fluid flow increases the static pull-in voltage of the cantilever CNTs by increasing the stiffness of the system, whereas for the CNTs with doubly clamped boundary conditions the static pull-in voltage is decreased because of a lowered stiffness. This can be attributed to the axial force that is induced by the fluid flow. For the cantilevered CNT, the axial force is tension because the free end does not apply any external force on the system. However, for the doubly clamped boundary conditions, the axial force is a compressive force because the clamped ends transform the axial force into a compression. According to the concepts of elasticity and vibration of continuous systems, the tensile force increases the natural frequency and buckling loads of the nanobeam, here the CNT, and the compressive force decreases both of them. In addition, the comparison reveals that the doubly clamped CNT deflects more than the cantilever CNT before the static pull-in. This can be related to the stiffer structure of the doubly clamped boundary conditions in comparison to the cantilever boundary conditions. This stiffer structure results in a very small deflection of the doubly clamped CNT under the vdW force before applying the electrostatic voltage.

**Figure 5:**
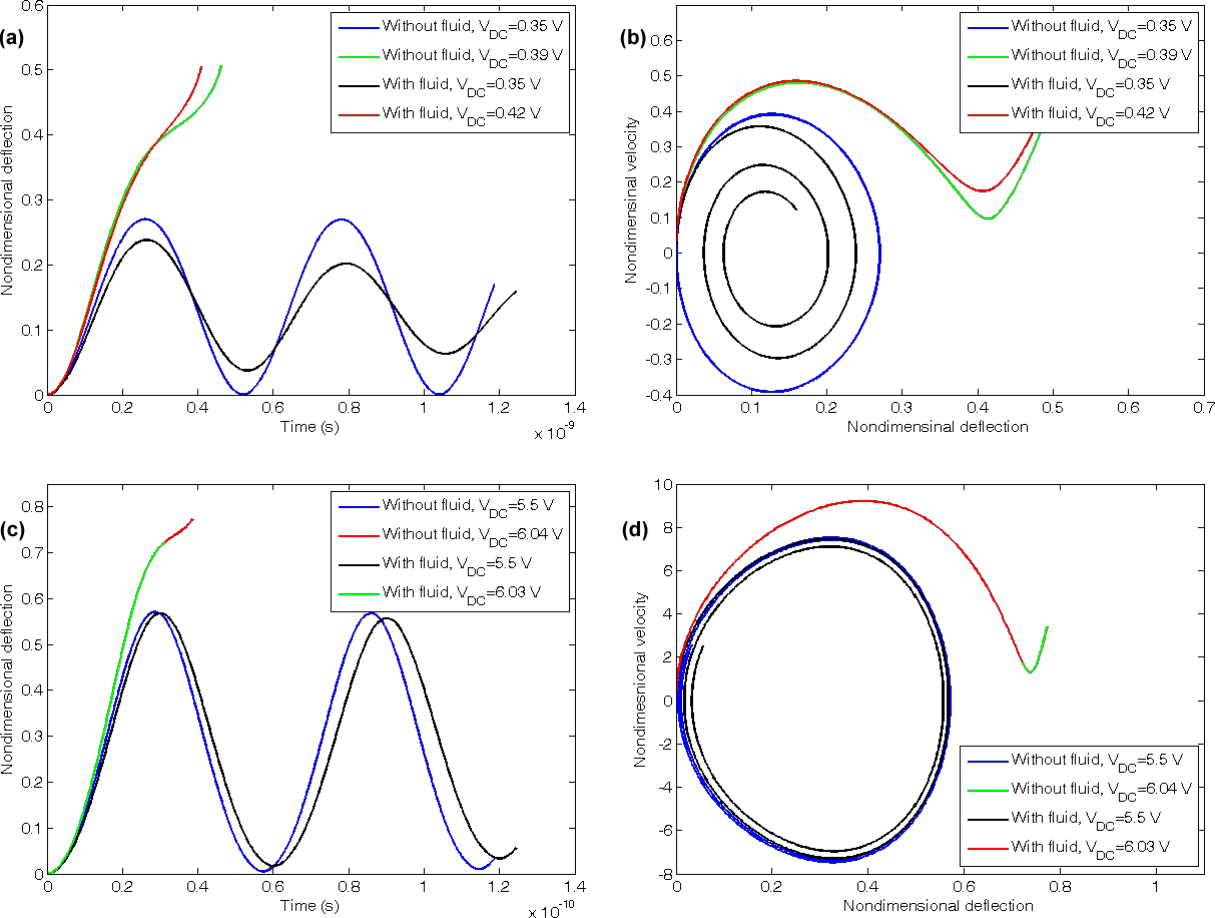
Vibration and phase plane of the (a), (b) cantilever, (c), (d) doubly clamped CNTs under step DC voltage.

The dynamic behavior of the cantilever and doubly clamped CNTs are illustrated in Figure 5. The viscosity is considered to be 1 mPa·s. The black and blue curves in the figures reveal that the CNT shows an oscillatory motion under the stepped DC voltage. The difference is that the CNT that convey a fluid show a damped harmonic motion whereas the fluid free CNT show an undamped motion. Hence, the fluid flow not only affects the stiffness of the system but also influences the damping properties. In addition, the green and red curves show that if the applied voltage exceeds a maximum limit, the CNT does not move harmonically anymore. This phenomenom that is corresponded to the saddle-node bifurcation is known as dynamic pull-in and the corresponding voltage is the dynamic pull-in voltage.

Since, the fluid flow influences both stiffness and damping properties of the CNT, it can be applied as a proper tool for controlling and positioning purposes. By using different parameters of the fluid flow, one can control the behaviors of the CNT for a special goal. In the following section the effects of the mentioned parameters on the static and dynamic pull-in voltages of the CNTs that convey fluids are given.

## Effects of changes in parameters of fluid flow

In this section, the effects of changes in the parameters of the fluid flow on the static and dynamic pull-in voltages of the CNT are investigated and the results of classical elasticity theory (CET) and MCST are compared. The outcomes are presented for both cantilever and doubly clamped boundary conditions. Some of the mentioned parameters do not affect the pull-in voltages. The fluid parameters are the same as presented in Table 1, unless stated explicitly otherwise or in the cases that their variation effects are investigated. In order to have a better view on the quality and quantity of the effects, the pull-in voltages are scaled with respect to the pull-in voltages obtained using the CET presented in Table 2 and corresponding to the fluid parameters considered in Figure 4 and Figure 5. The results are to be investigated for two representative low and high viscosity values, μ_low_ = 1 mPa·s and μ_low_ = 1 mPa·s, and the scaling of the pull-in voltages is done corresponding to these values.

**Table 2:** Reference pull-in voltages.

conditions	pull-in voltage	

	μ_low_ = 1 mPa·s	μ_low_ = 1 mPa·s

static pull-in of cantilever boundary conditions	0.39 V	1.9 V
static pull-in of doubly clamped boundary conditions	5.46 V	5.46 V
dynamic pull-in of cantilever boundary conditions	0.39 V	—
dynamic pull-in of doubly clamped boundary conditions	5.93 V	6.91 V

**Figure 6:**
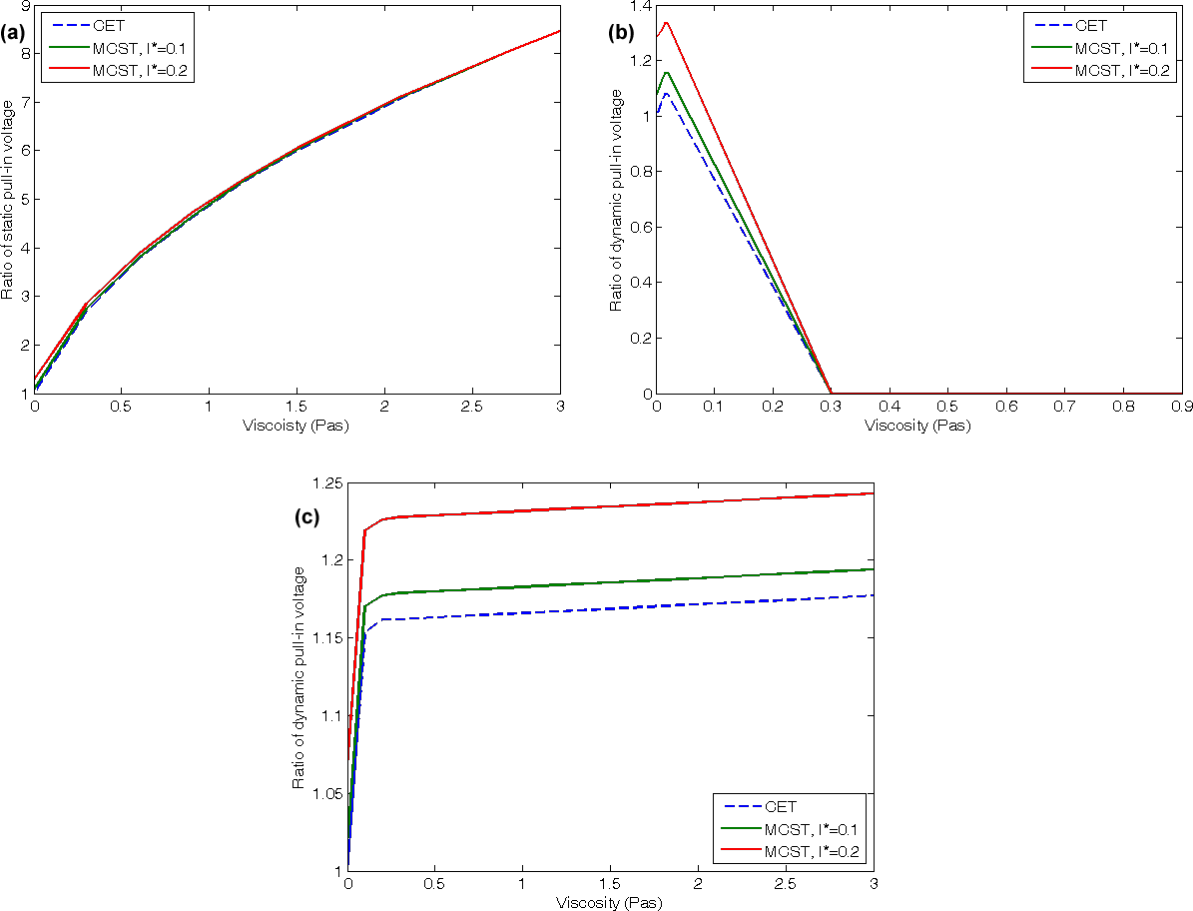
Effects of viscosity on (a) static pull-in voltages of the cantilevered CNT, (b) dynamic pull-in voltages of the cantilevered CNT, (c) dynamic pull-in voltages of the doubly clamped CNT.

Figure 6a illustrates the influences of fluid viscosity on the static pull-in voltages of the cantilevered CNT. The figure reveals that increasing the viscosity results in an increasing static pull-in voltage of the cantilevered CNT. In addition, it shows that higher length scale parameters lead to slightly higher static pull-in voltages of the mentioned system. However, the results corresponding to various length scale parameters approach each other with an increasing viscosity. Our calculations reveal that a variation in viscosity, at least in the ranges of the study in this paper, does not affect the static pull-in voltages of the doubly clamped CNTs. On the other hand, the dynamic pull-in voltages of cantilevered CNT as a function of the fluid viscosity are depicted in Figure 6b. With increasing viscosity the dynamic pull-in voltages of the cantilevered CNT raise up to a maximum value and then drop to zero voltage. This is the point at which the flutter instability occurs. The viscosity range before the voltage drop, μ < 0.02 Pa·s, covers the viscosity values of very common fluids such as water. In this range the system can be properly applied as a fluid viscometer in the dynamic mode as well as in the static mode. Figure 6c presents the effects of fluid viscosity on the dynamic pull-in voltages of the doubly clamped CNT. The dynamic pull-in voltages of the CNTs with doubly clamped boundary conditions first raise with a sharp slope. After a treshold value, the slope is reduced drastically but the dynamic pull-in voltages continue to increase. Similar to the static case, increasing the non-dimensional length scale parameter increases the dynamic pull-in voltages as well.

**Figure 7:**
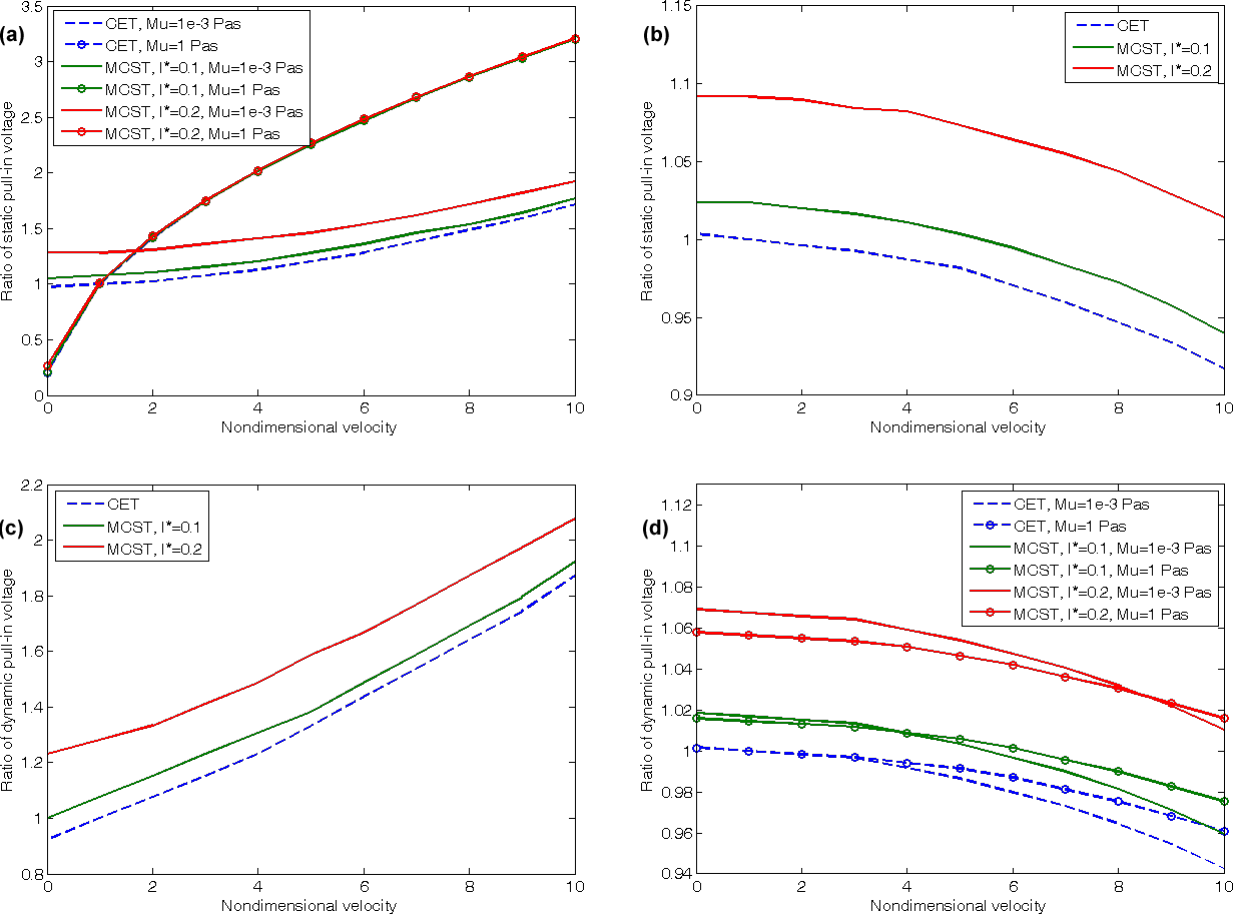
Effects of the fluid velocity on (a) the static pull-in voltage of cantilever CNT, (b) the static pull-in voltage of doubly clamped CNT, (c) the dynamic pull-in voltage of cantilever CNT, (d) the dynamic pull-in voltage of doubly clamped CNT.

Figure 7 illustrates the influences of the fluid velocity on the static and dynamic pull-in voltages. Figure 7a shows the effects on the static pull-in voltages of the cantilevered CNT for two viscosity values. Although, for both of them, the static pull-in voltage increases with increasing velocities, the variation is more intense for the higher viscosity. In addition, the higher the viscosity value, the more similar are the results for various length scales. Figure 7b illustrates the static pull-in voltages of the doubly clamped CNT as a function of the fluid velocity. Based on these results, one can conclude that, unlike for the cantilever boundary conditions, an increase of the fluid velocity reduces the static pull-in voltages of the CNTs with doubly clamped boundary conditions. As mentioned before, the viscosity variation does not influence the static pull-in voltages of the doubly clamped CNTs but the changes in the length scale parameter have direct effects on the pull-in voltages. The effects of varying the fluid velocity on the dynamic pull-in voltages are similar to the static pull-in voltages except for some points. The first point is that, as described before, the cantilevered CNT cannot be applied at high viscosity values due to the flutter instability. Hence, Figure 7c is presented only for the low viscosity. On the other hand, the dynamic pull-in voltages of the doubly clamped CNT, unlike the static pull-in, can be investigated for high viscosities as well as low values. In addition, changes in the values of the length scale parameter vary the pattern of variations with regard to the different viscosities in the dynamic pull-in of the CNTs with doubly clamped boundary conditions. In case of the CET that corresponds to a non-dimensional length scale parameter of zero, the higher viscosities result in higher pull-in voltages but for a parameter equaling 0.2, the lower viscosities generally lead to the higher pull-in voltages.

**Figure 8:**
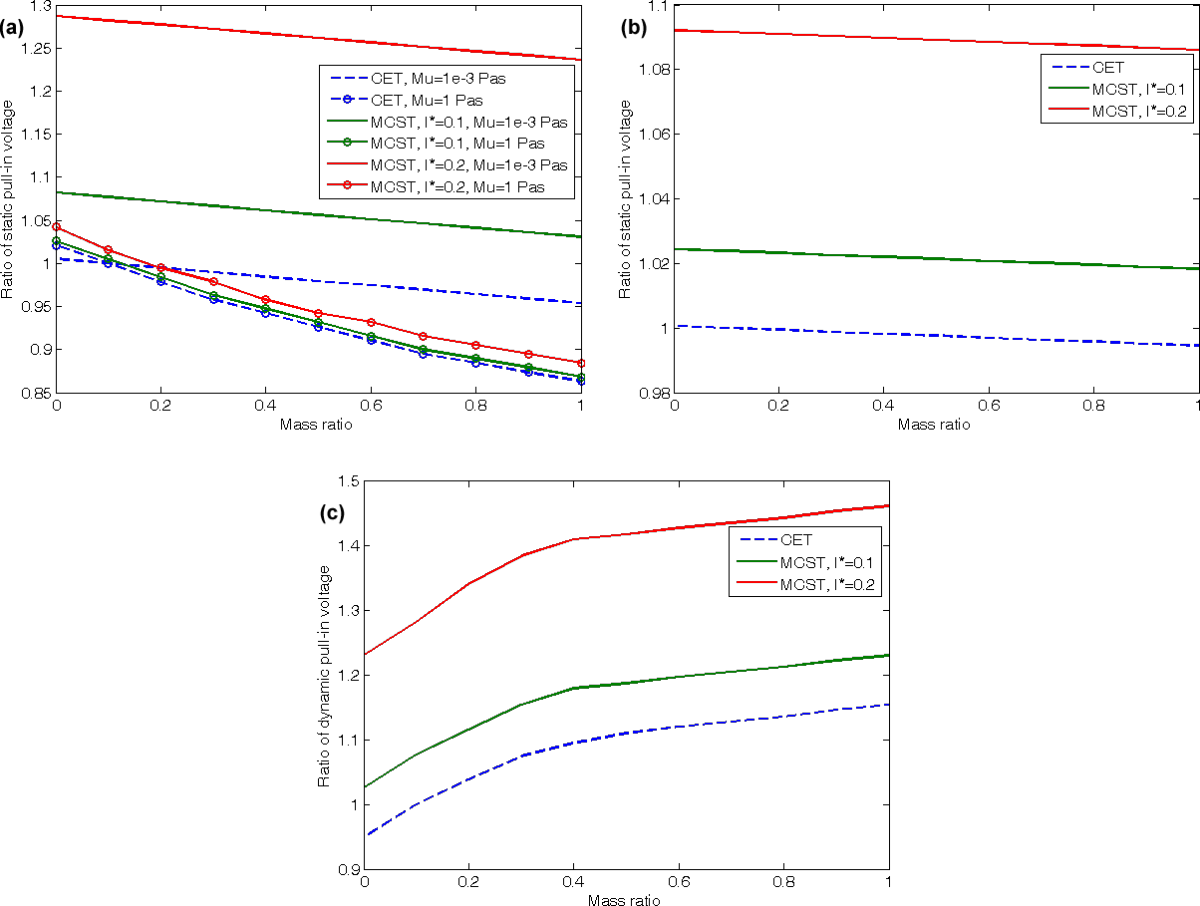
Effects of changes in the mass ratio on (a) the static pull-in voltages of cantilevered CNT, (b) the dynamic pull-in voltages of cantilevered CNT, (c) the static pull-in voltages of doubly clamped CNT.

The effects of changes in the mass ratio on the static and dynamic pull-in voltages of the CNT are studied in Figure 8. Figure 8a and Figure 8b illustrate the effects of increasing the mass ratio on the static pull-in voltages of the cantilever and doubly clamped CNTs, respectively. Increasing the mass ratio decreases the static pull-in voltages of the cantilevered CNT, while it increases the static pull-in voltages for the doubly clamped CNT. Increasing the viscosity would add to the mass ratio effects on the former. Moreover, increasing the non-dimensional length scale parameter increases the stiffness of the CNT and results in higher static pull-in voltages, similar to the previous cases.

A variation of the mass ratio does not affect the dynamic pull-in behavior of the cantilevered CNTs and only shows little effect on the dynamic pull-in voltages of the doubly clamped ones. The mass ratio increment decreases the dynamic pull-in voltages of the CNTs with doubly clamped boundary conditions.

**Figure 9:**
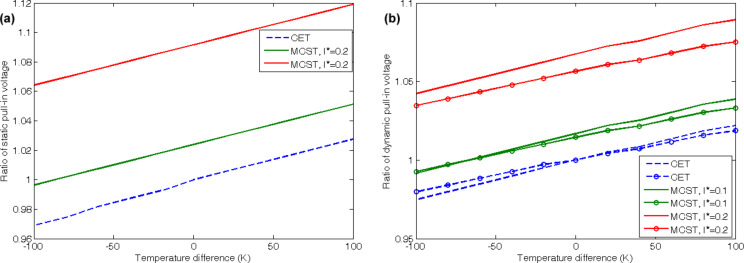
Effects of a variation in temperature on (a) the static, (b) the dynamic pull-in voltages of the doubly clamped CNTs.

Figure 9 depicts the influences of the temperature variation on the static and dynamic pull-in voltages of the doubly clamped CNT. A temperature difference of 0 K corresponds to 300 K. Both pull-in voltages increase with increasing temperature and length scale. An increase of to the length scale parameter increases also increases the slope of the curves. The main point related to the dynamic pull-in voltages is the effects of viscosity values. Similar to the effects of fluid velocity, for the results calculated from the CET, temperature increasing results in higher voltages for the CNTs that convey a fluid with high viscosity. On the contrary, temperature decreasing leads to lower pull-in voltages of the CNTs transporting a fluid of high viscosity. But for the stiffer structures that are obtained by application of MCST, the pull-in voltages for CNTs that convey low viscosity fluids dominate the voltages that are related to high viscosities.

Furthermore, our simulations reveal that a variation of the flow pressure does not, at least in the considered range of the parameters, affect the pull-in voltages of the CNTs. This can be attributed to this fact that a pressure variation does not impose remarkable changes on the stiffness of the system with the properties and dimensions considered here. In fact, the effects are so small that they can be neglected. In order to have a system sensitive to the fluid pressure, one would need to weaken the nanostructure by using larger lengths or smaller diameters of the CNT.

## Conclusion

In this paper, the applicability of the electrostatically actuated CNTs that convey fluids as nanosensors or nanoactuators was investigated. The model of an Euler–Bernoulli beam was applied in modeling the CNT by using MCST and the effects of the fluid flow were formulated by using the Navier–Stokes equations. The effects of fluid flow on the static and dynamic pull-in voltages of the CNTs under cantilever and doubly clamped boundary conditions were studied in detail. Certain flow parameters, i.e., fluid velocity, viscosity, mass and temperature were varied and the effects were scrutinized. The results showed various trends in the static and dynamic pull-in voltages of the CNTs. For example, an increase of fluid velocity increased the pull-in voltages of the cantilevered CNTs, whereas it decreased the pull-in voltages of the doubly clamped CNTs. All of the variations were justified in detail. In addition, the results revealed that the proposed system can be applied properly for the purposes mentioned above. The fluid flow through the CNT can be applied as control parameters for a desired behavior of the CNT by changing the stiffness and the damping of the system.

## Supporting Information

The Supporting Information features the comprehensive presentation of the mathematical formulae and expressions that are used in this study.

File 1 Mathematical formulae.

## References

[1] (2013). Nanotechnology - Definition and More from the Free Merriam-Webster Dictionary.

[2] Chandran S, Praveen G, Snima K S, Nair S V, Pavithran K, Chennazhi K, Lakshmanan V K (2013). Curr Drug Delivery.

[3] Liu J, Chen S, Lv L, Song L, Guo S, Huang S (2013). Curr Pharm Des.

[4] Karra N, Nassar T, Laenger F, Benita S, Borlak J (2013). Curr Cancer Drug Targets.

[5] Fakhrabadi M M S, Khani N, Omidvar R, Rastgoo A (2012). Comput Mater Sci.

[6] Fakhrabadi M M S, Amini A, Rastgoo A (2012). Comput Mater Sci.

[7] Fakhrabadi M M S, Norouzifard V, Dadashzadeh B, Allahverdizadeh A (2012). Solid State Commun.

[8] Kim J H, Yun S W, An B-K, Han Y D, Yoon S-J, Joo J, Park S Y (2013). Adv Mater.

[9] Fakhrabadi M M S, Khorasani P K, Rastgoo A, Ahmadian M T (2013). Solid State Commun.

[10] Fakhrabadi M M S, Rastgoo A, Ahmadian M T (2013). Fullerenes, Nanotubes, Carbon Nanostruct.

[11] Fakhrabadi M M S, Rastgoo A, Ahmadian M T (2013). J Comput Theor Nanosci.

[12] Hwang H J, Kang J W (2005). Physica E.

[13] Iijima S (1991). Nature.

[14] Dequesnes M, Rotkin S V, Aluru N R (2002). Nanotechnology.

[15] Dequesnes M, Aluru N R, Tang Z (2004). J Eng Mater Technol.

[16] Ke C, Espinosa H D (2005). J Appl Mech.

[17] Ke C, Espinosa H D, Pugno N (2005). J Appl Mech.

[18] Ke C-H, Pugno N, Peng B, Espinosa H D (2005). J Mech Phys Solids.

[19] Ouakad H M, Younis M I (2009). J Comput Nonlinear Dyn.

[20] Rasekh M, Khadem S E, Tatari M (2010). J Phys D: Appl Phys.

[21] Tuzun R E, Noid D W, Sumpter B G, Merkle R C (1996). Nanotechnology.

[22] Mirramezani M, Mirdamadi H R (2012). Physica E.

[23] Yoon J, Ru C Q, Mioduchowski A (2006). Int J Solids Struct.

[24] Wang L, Ni Q (2008). Comput Mater Sci.

[25] Wang L, Ni Q (2009). Mech Res Commun.

[26] Chang W-J, Lee H-L (2009). Phys Lett A.

[27] Solares S D, Blanco M, Goddard W A (2004). Nanotechnology.

[28] Grujicic M, Cao G, Roy W N (2005). Appl Surf Sci.

[29] Chen H Y, Liu Z F, Gong X G, Sun D Y (2011). Microfluid Nanofluid.

[30] Rinaldi S, Prabhakar S, Vengallatore S, Païdoussis M P (2010). Journal of Sound and Vibration.

