# Supplementary material for: Size-dependent characteristics of electrostatically actuated fluid-conveying carbon nanotubes based on modified couple stress theory
Source: Beilstein J Nanotechnol. 2013 Nov 20;4:771–80. doi: 10.3762/bjnano.4.88 (PMC3869342; doi:10.3762/bjnano.4.88)
Supplement: File 1 — Mathematical formulae. [file Beilstein_J_Nanotechnol-04-771-s001.pdf]

Supporting Information

for

Size-dependent characteristics of electrostatically actuated fluid-conveying carbon nanotubes based on modified couple stress theory

Mir Masoud Seyyed Fakhrabadi[*1,§], Abbas Rastgoo[2], Mohammad Taghi Ahmadian[3]

Address: [1]Karaj Branch, Islamic Azad University, Karaj, Iran, [2]School of Mechanical Engineering, College of Engineering, University of Tehran, Tehran, Iran and [3]Department of Mechanical Engineering, Sharif University of Technology, Tehran, Iran

Email: Mir Masoud Seyyed Fakhrabadi* - msfakhrabadi@gmail.com

* Corresponding author

[§] Tel/Fax: +98 935 5928477

Mathematical formulae

Mathematical formulae

The governing equation for the dynamic behavior of the CNT under the external load, which uses the modified couple stress theory (MCST), is presented in Equation 1.

$$(EI + GAl^2)\frac{\partial^4 w}{\partial x^4} - T^*\frac{\partial^2 w}{\partial x^2} + +c\frac{\partial w}{\partial t} = q_{fluid} + q_{elec} + q_{vdW} \tag{1}$$

where E, I, G, A, l, w, x, T^*, c and t at the left-hand side are, respectively, elastic modulus, moment of inertia, shear modulus, cross-sectional area, length scale parameter, deflection, axial coordinate, axial force, damping coefficient and time. In addition, q_{fluid}, q_{elec}, and q_{vdW} at the right-hand side denote the forces that result from the fluid flow, the electrostatic actuation and vdW interaction, respectively. The force due to the fluid flow can be obtained using the Navier–Stokes equation as below [1]:

$$q_{fluid} = \left[m_f v_{av}^2 + p^* A\right]\frac{\partial^2 w}{\partial x^2} - 2m_f v_{av}\frac{\partial^2 w}{\partial x \partial t} - \left(m_c + m_f\right)\frac{\partial^2 w}{\partial t^2} + \mu A_i \frac{\partial^3 w}{\partial x^2 \partial t} + \mu A_i v_{av}\frac{\partial^3 w}{\partial x^3} \tag{2}$$

where m_{f}, v_{av}, p^*, m_{c}, μ and A_{i} denote, respectively, fluid mass, fluid velocity, fluid pressure, mass of the CNT per length, fluid viscosity and fluid cross section. Inserting the above formula in Equation 1, we can write:

$$(EI + GAl^2)\frac{\partial^4 w}{\partial x^4} + \left[m_f v_{av}^2 + p^* A - T^* - N_t\right]\frac{\partial^2 w}{\partial x^2} + 2m_f v_{av}\frac{\partial^2 w}{\partial x \partial t} + \left(m_c + m_f\right)\frac{\partial^2 w}{\partial t^2} - \mu A_i \frac{\partial^3 w}{\partial x^2 \partial t} - \mu A_i v_{av}\frac{\partial^3 w}{\partial x^3} + c\frac{\partial w}{\partial t} = q_{elec} + q_{vdW} \tag{3}$$

where N_{t} is the axial force that results from the thermal variation and can be obtained from Equation 4 [2]:

$$N_t = -\frac{EA}{1-2\nu}\alpha_x \Delta T \tag{4}$$

where ν, α_x and ΔT are Poisson's ratio, the longitudinal thermal expansion coefficient and the temperature change, respectively. The axial force T^* is considered to be the mid-plane stretching effect equaling:

$$T^* = \left(\frac{EA}{2L}\int_0^L \left(\frac{\partial w}{\partial x}\right)^2\right)\frac{\partial^2 w}{\partial x^2} \tag{5}$$

All of the parameters $[p^*A - T^* - N_t]\frac{\partial^2 w}{\partial x^2}$ in the second term of Equation 3 equal zero for the cantilever boundary conditions. The maximum deflections of the cantilever and doubly clamped CNTs are at the tip or longitudinal center, respectively. The cantilever and doubly clamped boundary conditions, respectively, can be formulated as below:

$$\frac{\partial w(0,t)}{\partial x} = w(0,t) = \frac{\partial^2 w(L,t)}{\partial x^2} = \frac{\partial^3 w(L,t)}{\partial x^3} = 0 \tag{6}$$

$$\frac{\partial w(0,t)}{\partial x} = w(0,t) = \frac{\partial w(L,t)}{\partial x} = w(L,t) = 0 \tag{7}$$

where, L is the length of the CNT.

The considered model, i.e., the continuum model, has been applied in different papers [3-11]. All of them considered continuum mechanics for the fluid flow through the nanotube in their researches. The CNT studied in this paper has a diameter larger than 1 nm. Thus, the fluid flow through it can be investigated via continuum mechanics. On the other hand, this study can be easily generalized for the CNTs with larger dimensions based on the requirements of the system.

The electrostatic force on the right-hand side of Equation 3 is presented as [12]:

$$q_{elec} = \frac{\pi \varepsilon_0 V^2}{\sqrt{(G_0 - w)(G_0 - w + 2R)}\ \mathrm{arccosh}^2\left(1 + \frac{G_0 - w}{R}\right)} \tag{8}$$

where ε_0, V, R and G_0, respectively, represent the electrical permittivity, voltage, radius of the CNT and initial gap.

In addition, the vdW force on the right-hand side of Equation 3 can be formulated as below [12]:

$$q_{vdw} = \frac{\pi^2 c_6 \sigma^2}{2} \sum_{n=1}^{N_G} \frac{R\begin{pmatrix} 8(G_0 + (n-1)d - w)^4 + 32(G_0 + (n-1)d - w)^3 R \\ +72(G_0 + (n-1)d - w)^2 R^2 + 80(G_0 + (n-1)d - w)R^3 + 35R^4 \end{pmatrix}}{(G_0 + (n-1)d - w)^{\frac{9}{2}} (G_0 + (n-1)d - w + 2R)^{\frac{9}{2}}} \tag{9}$$

where c_6 and σ^2 are the parameters of the Lennard-Jones potential that describes the vdW force and N_G represents number of graphene sheets.

Solution of the governing equations

By introducing the following non-dimensional parameters, we can non-dimensionalize the terms of the governing equations.

$$\bar{w} = \frac{w}{G_0}, \bar{x} = \frac{x}{L}, \bar{R} = \frac{R}{G_0}, \bar{d} = \frac{d}{G_0}, \bar{t} = \frac{t}{t^* = \sqrt{\frac{(m_c + m_f)\, L^4}{EI}}} \tag{10}$$

Inserting these non-dimensional parameters in Equation 3, Equation 8 and Equation 9 we get:

$$\left(1 + \frac{\alpha_0 l^2}{(d_o^2 + d_i^2)}\right)\frac{\partial^4 \bar{w}}{\partial \bar{x}^4} + \alpha_1 \frac{\partial^2 \bar{w}}{\partial \bar{x}^2} + \beta_1 \frac{\partial^2 \bar{w}}{\partial \bar{x}\partial \bar{t}} + \gamma_1 \frac{\partial^2 \bar{w}}{\partial \bar{t}^2} - \delta_1 \frac{\partial^3 \bar{w}}{\partial \bar{x}^2 \partial \bar{t}} - \xi_1 \frac{\partial^3 \bar{w}}{\partial \bar{x}^3} + \zeta_1 \frac{\partial \bar{w}}{\partial \bar{t}} = \bar{q}_{elec} + \bar{q}_{vdW} \tag{11}$$

where d_o and d_i are the outer and inner diameters of the CNT and

$$\alpha_0 = \frac{16\mu}{E}$$

$$\alpha_1 = \left[m_f v_{av}^2 + p^* A - \left(\alpha \int_0^1 \left(\frac{\partial \bar{w}}{\partial \bar{x}}\right)^2 d\bar{x}\right) - N_t\right]\frac{L^2}{EI}$$

$$\alpha = \frac{EAG_0^2}{2L^2}$$

$$\beta_1 = \frac{2m_f v_{av} L^3}{EIt^*}$$

$$\gamma_1 = \left(m_c + m_f\right)\frac{L^4}{EI{t^*}^2}$$

$$\delta_1 = \frac{\mu A_i L^2}{t^* EI}$$

$$\xi_1 = \frac{\mu A_i v_{av} L}{EI} \qquad (12)$$

$$\zeta_1 = \frac{cL^4}{EIt^*}$$

$$\beta = \frac{\pi \varepsilon_0 L^4}{EIG_0^2}$$

$$\bar{q}_{elec} = \frac{\beta V^2}{\sqrt{(1-\bar{w})(1-\bar{w}+2\bar{R})}\,\mathrm{arccosh}^2\left(1+\frac{1-\bar{w}}{\bar{R}}\right)}$$

$$\gamma = \frac{\pi^2 c_6 \sigma^2 L^4}{2EIG_0^5}$$

$$\bar{q}_{vdW} = \gamma \sum_{n=1}^{N_G} \frac{\bar{R}\begin{pmatrix} 8\left(1+(n-1)\bar{d}-\bar{w}\right)^4 + 32\left(1+(n-1)\bar{d}-\bar{w}\right)^3\bar{R} \\ +72\left(1+(n-1)\bar{d}-\bar{w}\right)^2\bar{R}^2 + 80\left(1+(n-1)\bar{d}-\bar{w}\right)\bar{R}^3 + 35\bar{R}^4 \end{pmatrix}}{\left(1+(n-1)\bar{d}-\bar{w}\right)^{\frac{9}{2}}\left(1+(n-1)\bar{d}-\bar{w}+2\bar{R}\right)^{\frac{9}{2}}}$$

As mentioned before α, p^* and N_t equal zero for the cantilever boundary conditions.

Static deflection under DC voltage

For the static actuation of the nanosystem, the terms of Equation 11 that contain time derivatives are set to zero. Thus, the partial derivatives transform to the ordinary ones. Hence, the governing equation for the static deflection of the CNT under a gradually increasing DC voltage is presented in Equation 13.

$$\left(1+\frac{\alpha_0 l^2}{(d_o^2+d_i^2)}\right)\frac{d^4\bar{w}}{d\bar{x}^4}+\alpha_1\frac{d^2\bar{w}}{d\bar{x}^2}-\xi_1\frac{d^3\bar{w}}{d\bar{x}^3}=\bar{q}_{elec}+\bar{q}_{vdW}\ =F(V,w) \tag{13}$$

The equation above is solved by using the step-by-step linearization method (SSLM) [13]. This is an effective approach to solve the nonlinear governing equations of the MEMS and NEMS and it has been applied in various studies [14-17]. For the current case, we apply SSLM twice: The first time only for the vdW force and the next time for both vdW and electrostatic forces. The first one is required to obtain the equilibrium position of the CNT under only the vdW force before a DC voltage is applied. Considering λ_i in the range [0,1], the first SSLM is applied. Hence, for the *i*th step Equation 13 can be rewritten as follows:

$$\left(1+\frac{\alpha_0 l^2}{(d_o^2+d_i^2)}\right)\frac{d^4\bar{w}_i}{d\bar{x}^4}+\alpha_1\frac{d^2\bar{w}_i}{d\bar{x}^2}-\xi_1\frac{d^3\bar{w}_i}{d\bar{x}^3}=\left(\bar{q}_{vdW}(\bar{w}_i)\right) \tag{14}$$

It can be formulated for the $(i+1)$th step as below:

$$\left(1+\frac{\alpha_0 l^2}{(d_o^2+d_i^2)}\right)\frac{d^4\bar{w}_{i+1}}{d\bar{x}^4}+\alpha_1\frac{d^2\bar{w}_{i+1}}{d\bar{x}^2}-\xi_1\frac{d^3\bar{w}_{i+1}}{d\bar{x}^3}=\lambda_{i+1}\left(\bar{q}_{vdW}(\bar{w}_{i+1})\right) \tag{15}$$

Substituting $\bar{w}_{i+1}=\bar{w}_i+\bar{\varphi}_i$ and $\lambda_{i+1}=\lambda_i+\delta\lambda$ in Equation 15, subtracting Equation 14 from Equation 15 and using a Taylor expansion for the right-hand side of Equation 15, we have:

$$\left(1+\frac{\alpha_0 l^2}{(d_o^2+d_i^2)}\right)\frac{d^4\bar{\varphi}_i}{d\bar{x}^4}+\alpha_1\frac{d^2\bar{\varphi}_i}{d\bar{x}^2}-\xi_1\frac{d^3\bar{\varphi}_i}{d\bar{x}^3}-\lambda_i\frac{\partial\bar{q}_{vdW}}{\partial\bar{w}}\bigg|_{\bar{w}_i}\bar{\varphi}_i=\left(\bar{q}_{vdW}(\bar{w}_i)\right)\delta\lambda \tag{16}$$

where

$$\frac{\partial \bar{q}_{vdW}}{\partial \bar{w}} = \gamma \sum_{n=1}^{N_G} \left[\frac{\bar{R}\begin{pmatrix} -32\left(1+(n-1)\bar{d}-\bar{w}\right)^3 - 96\left(1+(n-1)\bar{d}-\bar{w}\right)^2 \bar{R} \\ -144\left(1+(n-1)\bar{d}-\bar{w}\right)\bar{R}^2 - 80\bar{R}^3 \end{pmatrix}}{\left(1+(n-1)\bar{d}-\bar{w}\right)^{\frac{9}{2}}\left(1+(n-1)\bar{d}-\bar{w}+2\bar{R}\right)^{\frac{9}{2}}} \right. \tag{17}$$

$$+\frac{9}{2}\left(\frac{1}{\left(1+(n-1)\bar{d}-\bar{w}\right)^{\frac{11}{2}}\left(1+(n-1)\bar{d}-\bar{w}+2\bar{R}\right)^{\frac{9}{2}}}\right.$$

$$\left.+\frac{1}{\left(1+(n-1)\bar{d}-\bar{w}\right)^{\frac{9}{2}}\left(1+(n-1)\bar{d}-\bar{w}+2\bar{R}\right)^{\frac{11}{2}}}\right)\left(\bar{R}\left(8\left(1\right.\right.\right.$$

$$+(n-1)\bar{d}-\bar{w}\Big)^4 + 32\left(1+(n-1)\bar{d}-\bar{w}\right)^3\bar{R}$$

$$\left. +72\left(1+(n-1)\bar{d}-\bar{w}\right)^2\bar{R}^2\Big) + 80\left(1+(n-1)\bar{d}-\bar{w}\right)\bar{R}^3 + 35\bar{R}^4\Big) \right]$$

In order to obtain the equilibrium position of the carbon nanotube the Galerkin method is applied to Equation 16. A detailed description of this method is to be discussed in the following lines. The deflection obtained in this step is added to the deflection to be calculated in the next application of the SSLM.

Now, the second application of the SSLM is going to be used. The voltage and deflection at the *k*th step are V_k and w_k, and at the (*k*+1)th step they are V_{k+1} and w_{k+1}. By subtracting the *k*th step from the (*k*+1)th step, we can write:

$$V_{k+1} - V_k = dV; \qquad w_{k+1} - w_k = \psi \tag{18}$$

For the *k*th step Equation 16 can be rewritten as Equation 19:

$$\left(1+\frac{\alpha_0 l^2}{(d_o^2+d_i^2)}\right)\frac{d^4\overline{w}_k}{d\bar{x}^4}+\alpha_1\frac{d^2\overline{w}_k}{d\bar{x}^2}-\xi_1\frac{d^3\overline{w}_k}{d\bar{x}^3}=F(V_k,w_k) \tag{19}$$

Also, for the (k+1)th step Equation 16 can be rewritten as Equation 20:

$$\left(1+\frac{\alpha_0 l^2}{(d_o^2+d_i^2)}\right)\frac{d^4\overline{w}_{k+1}}{d\bar{x}^4}+\alpha_1\frac{d^2\overline{w}_{k+1}}{d\bar{x}^2}-\xi_1\frac{d^3\overline{w}_{k+1}}{d\bar{x}^3}=F(V_{k+1},w_{k+1}) \tag{20}$$

It is worth noting that for very small deviations, $F(V_{k+1},w_{k+1})$, can be expanded into a two-dimensional Taylor series as follows:

$$F(V_{k+1},w_{k+1})=F(V_k,w_k)+\left.\frac{\partial F}{\partial V}\right]_{V_k,w_k}\delta V+\left.\frac{\partial F}{\partial w}\right]_{V_k,w_k}\delta w \tag{21}$$

where

$$\frac{\partial F}{\partial V}=\frac{\partial\bar{q}_{elec}}{\partial V}+\frac{\partial\bar{q}_{vdW}}{\partial V}\quad\text{and}\quad\frac{\partial F}{\partial\overline{w}}=\frac{\partial\bar{q}_{elec}}{\partial\overline{w}}+\frac{\partial\bar{q}_{vdW}}{\partial\overline{w}}$$

and

$$\begin{aligned}
&\frac{\partial\bar{q}_{elec}}{\partial V}=\frac{2\beta V}{\sqrt{(1-\overline{w})(1-\overline{w}+2\bar{R})}\,\mathrm{arccosh}^2\left(1+\frac{1-\overline{w}}{\bar{R}}\right)}\\
&\frac{\partial\bar{q}_{vdW}}{\partial V}=0\\
&\frac{\partial\bar{q}_{elec}}{\partial\overline{w}}\\
&=\frac{1}{2}\beta V^2\left(\frac{1}{(1-\overline{w})^{\frac{3}{2}}\sqrt{(1-\overline{w}+2\bar{R})}\,\mathrm{arccosh}^2\left(1+\frac{1-\overline{w}}{\bar{R}}\right)}\right.\\
&\left.+\frac{1}{\sqrt{1-\overline{w}}(1-\overline{w}+2\bar{R})^{\frac{3}{2}}\,\mathrm{arccosh}^2\left(1+\frac{1-\overline{w}}{\bar{R}}\right)}\right)\\
&+\frac{2\beta V^2}{\bar{R}\sqrt{1-\overline{w}}\sqrt{(1-\overline{w}+2\bar{R})}\sqrt{\frac{1-\overline{w}}{\bar{R}}}\sqrt{2+\frac{1-\overline{w}}{\bar{R}}}\,\mathrm{arccosh}^3\left(1+\frac{1-\overline{w}}{\bar{R}}\right)}
\end{aligned} \tag{22}$$

and $\frac{\partial\bar{q}_{vdW}}{\partial\overline{w}}$ is obtained from Equation 17.

By subtracting the kth step from the (k+1)th step, we get:

$$\left(1+\frac{\alpha_0 l^2}{(d_o^2+d_i^2)}\right)\frac{d^4\psi}{d\bar{x}^4}+\alpha_1\frac{d^2\psi}{d\bar{x}^2}-\xi_1\frac{d^3\psi}{d\bar{x}^3}-\left(\frac{\partial\bar{q}_{elec}}{\partial\bar{w}}+\frac{\partial\bar{q}_{vdW}}{\partial\bar{w}}\right)\Bigg]_{V_k,\bar{w}_k}\psi = \frac{\partial\bar{q}_{elec}}{\partial V}\Bigg]_{V_k,\bar{w}_k} dV \qquad (23)$$

Equation 23 is the final governing equation for the static deflection of the CNT under a static DC actuation. To solve it, we use the expansion theory as given below [18]:

$$\psi(x)=\sum_{j=1}^{N} a_j\varphi_j(x) \qquad (24)$$

The shape modes that correspond to the cantilever and doubly clamped boundary conditions are presented in Equation 25 and Equation 26, respectively [19].

$$\varphi(x)=\cosh\mu_{sm}\bar{x}-\cos\mu_{sm}\bar{x} - \frac{(\cosh\mu_{sm}+\cos\mu_{sm})}{(\sinh\mu_{sm}+\sin\mu_{sm})}(\sinh\mu_{sm}\bar{x}-\sin\mu_{sm}\bar{x}), \qquad \mu_{sm1}=1.875 \qquad (25)$$

$$\varphi(x)=\cosh\lambda_{sm}\bar{x}-\cos\lambda_{sm}\bar{x} - \frac{(\cosh\lambda_{sm}-\cos\lambda_{sm})}{(\sinh\lambda_{sm}-\sin\lambda_{sm})}(\sinh\lambda_{sm}\bar{x}-\sin\lambda_{sm}\bar{x}), \qquad \lambda_{sm1}=4.73 \qquad (26)$$

Only its first mode shape appears for the static deflection of the CNT, therefore we only consider the first one.

$$\psi(x)=a\varphi(x) \qquad (27)$$

Substituting Equation 27 in Equation 23, we have:

$$a\left(1+\frac{\alpha_0 l^2}{(d_o^2+d_i^2)}\right)\frac{d^4\varphi}{d\bar{x}^4}+\alpha_1 a\varphi''-\xi_1 a\frac{d^3\varphi}{d\bar{x}^3}-a\left(\frac{\partial\bar{q}_{elec}}{\partial\bar{w}}+\frac{\partial\bar{q}_{vdW}}{\partial\bar{w}}\right)\Bigg]_{V_k,\bar{w}_k}\varphi = \frac{\partial\bar{q}_{elec}}{\partial V}\Bigg]_{V_k,\bar{w}_k} dV \qquad (28)$$

By applying the Galerkin method, the governing equation to the static deflection of the CNT under a DC voltage is obtained as follows:

$$a(k_{1s} + \alpha_1 k_{2s} - \xi_1 k_{3s} - k_{4s}) = \int_0^1 \frac{\partial \bar{q}_{elec}}{\partial V}\Big]_{V_k,\bar{w}_k} \varphi dV d\bar{x} \tag{29}$$

where

$$k_{1s} = \left(1 + \frac{\alpha_0 l^2}{(d_o^2 + d_i^2)}\right)\int_0^1 \frac{d^4\varphi}{d\bar{x}^4}\varphi d\bar{x}$$

$$k_{2s} = \int_0^1 \frac{d^2\varphi}{d\bar{x}^2}\varphi d\bar{x}$$

$$k_{3s} = \int_0^1 \frac{d^3\varphi}{d\bar{x}^3}\varphi d\bar{x} \tag{30}$$

$$k_{4s} = \int_0^1 \left(\frac{\partial \bar{q}_{elec}}{\partial \bar{w}} + \frac{\partial \bar{q}_{vdW}}{\partial \bar{w}}\right)\Big]_{V_k,\bar{w}_k} \varphi^2 d\bar{x}$$

Dynamic behavior under stepped DC

In order to solve the governing equation for the dynamic behavior of the CNTs under electrostatic actuation, the expansion theory is applied to Equation 9 as follows [13]:

$$\psi(x) = \sum_{j=1}^{N} T_j(t)\varphi_j(x) \tag{31}$$

The first mode shape is considered in the investigation of the desired behavior. To simplify the solution procedure, the deflection on the right-hand side of Equation 9 in each step is considered same as the deflection in the previous step.

$$T\left(1 + \frac{\alpha_0 l^2}{(d_o^2 + d_i^2)}\right)\frac{d^4\varphi}{d\bar{x}^4} + \alpha_1 T\varphi'' + \beta_1 \dot{T}\varphi' + \gamma_1 \ddot{T}\varphi - \delta_1 \dot{T}\varphi'' - \xi_1 T\frac{d^3\varphi}{d\bar{x}^3} + \zeta_1 \dot{T}\varphi = \bar{q}_{elec} + \bar{q}_{vdW} \tag{32}$$

Applying the Galerkin method to the above equation, we get:

$$T(k_{1d} + \alpha_1 k_{2d} - \xi_1 k_{3d}) + \dot{T}(\beta_1 k_{4d} - \delta_1 k_{5d} + \zeta_1 k_{6d}) + \gamma_1 k_{7d} \ddot{T} \tag{33}$$

$$= \int_0^1 \bar{q}_{elec} \varphi d\bar{x} + \int_0^1 \bar{q}_{vdW} \, \varphi d\bar{x}$$

where

$$k_{1d} = k_{1s}$$

$$k_{2d} = k_{2s}$$

$$k_{3d} = k_{3s}$$

$$k_{4d} = \int_0^1 \varphi' \varphi d\bar{x} \tag{34}$$

$$k_{5d} = \int_0^1 \varphi'' \varphi d\bar{x}$$

$$k_{6d} = k_{7d} = \int_0^1 \varphi^2 d\bar{x}$$

Equation 34 is solved by using the 4th order Runge–Kutta method in order to obtain the dynamic behavior of the system and also to investigate the effects of fluid flow through the CNT.

The nondimensional length scale parameter of the MCST that will be applied in the following section is defined as below:

$$l^* = \sqrt{\frac{\alpha_0 l^2}{(d_o^2 + d_i^2)}} \tag{35}$$

References

1. Wang, L.; Ni, Q. *Mech. Res. Commun.* **2009,** *36,* 833–837.
2. Wang, L.; Ni, Q.; Li, M.; Qian, Q. *Physica E* **2008,** *40,* 3179–3182.
3. Kuang, Y. D.; He, X. Q.; Chen, C. Y.; Li, G. Q. *Comput. Mater. Sci.* **2009,** *45,* 875–880.
4. Ghavanloo, E.; Fazelzadeh, S. A. *Physica E* **2011,** *44,* 17–24.
5. Yoon, J.; Ru, C. Q.; Mioduchowski, A. *International Journal of Solids and Structures* **2006**, *43*, 3337–3349.
6. Wang, L.; Ni, Q. *Comput. Mater. Sci.* **2008,** *43,* 399–402.
7. Ghavanloo, E.; Daneshmand, F.; Rafiei, M. *Physica E* **2010,** *42,* 2218–2224.
8. Lee, H. L.; Chang, W. J. *Physica E* **2009,** *41,* 529–532.
9. Soltani, P.; Taherian, M. M.; Farshidianfar, A. *J. Phys. D: Appl. Phys.* **2010,** *43,* 425401.
10. Zhen, Y. X.; Fang, B.; Tang, Y. *Physica E* **2011,** *44,* 379–385.
11. Zhen, Y.; Fang, B. *Comput. Mater. Sci.* **2010,** *49,* 276–282.
12. Dequesnes, M.; Rotkin, S. V.; Aluru, N. R. *Nanotechnology* **2002,** *13,* 120–131.
13. Rezazadeh, G.; Tahmasebi, A.; Zubstov, M. *Microsyst. Technol.* **2006,** *12*, 1163–1170.
14. Motallebi, A.; Fathalilou, M.; Rezazadeh, G. *Acta Mechanica Solida Sinica (Chinese Edition)* **2012,** *25*, 627–637.
15. Mobki, H.; Rezazadeh, G.; Sadeghi, M.; Vakili-Tahami, F.; Fakhrabadi M. M. S. *International Journal of Non-Linear Mechanics* **2013,** *48*, 78–85.
16. Rezazadeh, G.; Fathalilou, M.; Sadeghi, M. *Sensing and Imaging: An International Journal* **2011,** *12,* 117–131.
17. Talebian, S.; Rezazadeh, G.; Fathalilou, M.; Toosi, B. *Mechatronics* **2010,** *20,* 666–673.
18. Younis, M. I.; Nayfeh, A. H. *Nonlinear Dyn.* **2003,** *31*, 91–117.
19. Mojahedi, M.; Moghimi Zand, M.; Ahmadian, M. T. *Applied Mathematical Modelling* **2010,** *34*, 1032–1041.
